# Woven Endobridge device for ruptured vs. unruptured aneurysms: insights from the WorldWideWEB study

**DOI:** 10.1007/s00234-026-04032-6

**Published:** 2026-05-18

**Authors:** Franja Dugar, Muhammed Amir Essibayi, Hamza Adel Salim, Basel Musmar, Nimer Adeeb, Mahmoud Dibas, Yan-Lin Li, Oktay Algin, Sherief Ghozy, Sovann V. Lay, Adrien Guenego, Leonardo Renieri, Joseph Carnevale, Guillaume Saliou, Panagiotis Mastorakos, Eimad Shotar, Markus Möhlenbruch, Michael Kral, Charlotte Chung, Mohamed M. Salem, Ivan Lylyk, Nicole M. Cancelliere, Jose Danilo Bengzon Diestro, Paul M. Foreman, Hamza Shaikh, Vedran Župančić, Muhammad U. Hafeez, Joshua Catapano, Muhammad Waqas, Giyas Ayberk, Muhammet Arslan, James D. Rabinov, Onur Ergun, Julian Maingard, Clemens M. Schirmer, Mariangela Piano, Anna L. Kühn, Caterina Michelozzi, Robert M. Starke, Ameer Hassan, Mark Ogilvie, Jesse Jones, Waleed Brinjikji, Fulvio Zaccagna, James T. Grist, Marios Psychogios, Marie T. Nawka, Jens Fiehler, Thi Dan Linh Nguyen-Kim, Bryan Pukenas, Jan-Karl Burkhardt, Thien Huynh, Juan Carlos Martinez-Gutierrez, Sunil A. Sheth, Diana Slawski, Rabih G. Tawk, Benjamin Pulli, Boris Lubicz, Pietro Panni, Ajit S. Puri, Guglielmo Pero, Eytan Raz, Christoph J. Griessenauer, Hamed Asadi, Adnan Siddiqui, Elad I Levy, Neil Haranhalli, David Altschul, Andrew F. Ducruet, Robert W. Regenhardt, Christopher J. Stapleton, Peter Kan, Vladimir Kalousek, Pedro Lylyk, Srikanth Boddu, Jared Knopman, Stavropoula I. Tjoumakaris, Hugo H. Cuellar-Saenz, Pascal M. Jabbour, Frédéric Clarençon, Nicola Limbucci, Tufail Patankar, Vitor Mendes Pereira, Aman B. Patel, Mohammad Almohammad, Andre Kemmling, Luca Scarcia, Adam A. Dmytriw, Peter B. Sporns

**Affiliations:** 1 Department of Radiology, Neuroradiology and Nuclear Medicine, Triemli Hospital, Zurich, Switzerland; 2 Department of Neurosurgery, Montefiore Medical Center, The Bronx, United States; 3 Department of Neuroradiology, The University of Texas MD Anderson Cancer Center, Houston, United States; 4 Department of Radiology, Johns Hopkins Bayview Medical Center, Baltimore, United States; 5 Department of Neurosurgery and Interventional Neuroradiology, Louisiana State University Health Sciences Center Shreveport, Shreveport, United States; 6 Nuffield Department of Surgical Sciences, Medical Sciences Division, University of Oxford, Oxford, United Kingdom; 7 Medical Faculty, Department of Radiology, Ankara University, Ankara, Turkey; 8 Departments of Radiology and Neurosurgery, Mayo Clinic, Rochester, United States; 9 Department of Neuroradiology, Centre Hospitalier Universitaire de Toulouse, Toulouse, France; 10 Department of Neuroradiology, Erasmus Hospital, Brussels, Belgium; 11 Department of Neuroradiology, Azienda Ospedaliero-Universitaria Careggi, Florence, Italy; 12 Department of Neurosurgery and Neuroradiology, NewYork–Presbyterian Hospital, New York, United States; 13 Department of Neuroradiology, University Hospital of Lausanne, Lausanne, Switzerland; 14 Department of Neurosurgery, Thomas Jefferson University Hospital, Philadelphia, United States; 15 Department of Neuroradiology, Pitié-Salpêtrière Hospital, Paris, France; 16 Department of Neuroradiology, University Hospital Heidelberg, Heidelberg, Germany; 17 Department of Neurosurgery, Christian Doppler Klinik, Salzburg, Austria; 18 Departments of Radiology & Neurosurgery, New York University Langone Medical Center, New York, United States; 19 Department of Neurosurgery, University of Pennsylvania, Philadelphia, United States; 20 Department of Neuroradiology, Clínica La Sagrada Familia, Buenos Aires, Argentina; 21 Neurovascular Centre, Divisions of Therapeutic Neuroradiology and Neurosurgery, St. Michael’s Hospital, Toronto, Canada; 22 Department of Neurosurgery, Orlando Health Neuroscience and Rehabilitation Institute, Orlando, United States; 23 Department of Radiology & Neurosurgery, Cooper University Hospital, Camden, United States; 24 Department of Neuroradiology, Sisters of Charity Hospital, Zagreb, Croatia; 25 Department of Neurosurgery, Barrow Neurological Institute, Phoenix, United States; 26 Department of Neurosurgery, University at Buffalo, State University of New York, Buffalo, United States; 27 Department of Neurosurgery, Ankara Yıldırım Beyazıt University, Ankara, Turkey; 28 Radiology Department, Pamukkale University, Denizli, Turkey; 29 Neuroendovascular Program, Massachusetts General Hospital, Boston, United States; 30 Memorial Hospital, Radiology Department, Üsküdar University, Skutari, Turkey; 31 Department of Neuroradiology, Austin Health, Melbourne, Australia; 32 Department of Neurosurgery and Radiology, Geisinger Medical Center, Danville, United States; 33 Department of Neuroradiology, Ospedale Niguarda Ca’ Granda, Milan, Italy; 34 Department of Neuroradiology, UMass Memorial Medical Center, Worcester, United States; 35 Department of Neuroradiology, IRCCS Ospedale San Raffaele, Milan, Italy; 36 Department of Neurosurgery, University of Miami, Florida, United States; 37 Department of Neuroradiology, Valley Baptist Medical Center, Harlingen, United States; 38 Departments of Neurosurgery and Radiology, University of Alabama at Birmingham, Birmingham, United States; 39 Oxford Centre for Clinical Magnetic Resonance (OCMR), Division of Cardiovascular Medicine, Radcliffe Department of Medicine, University of Oxford, Oxford, United Kingdom; 40 Department of Neuroradiology, University Hospital of Basel, Basel, Switzerland; 41 Department of Diagnostic and Interventional Neuroradiology, University Medical Center Hamburg-Eppendorf, Hamburg, Germany; 42 Department of Neuroradiology, The University of Texas Health Science Center at Houston, Houston, United States; 43 Department of Radiology, Division of Neuroimaging and Neurointervention, Stanford University, Stanford, United States; 44 Interventional Neurovascular Unit, Azienda Ospedaliero-Universitaria Careggi, Florence, Italy; 45 Department of Neuroradiology, Barking, Havering And Redbridge University Hospitals NHS Trust, London, United Kingdom; 46 Department of Neuroradiology, Universitätsklinikum Gießen und Marburg, Giessen, Germany; 47 Department of Neuroradiology, Hôpitaux Universitaires Henri-Mondor, Créteil, France; 48 Neurointerventional & Neuroanalytics Collaboration (NAN-C), University of Toronto, Toronto, Canada; 49 Department of Neuroradiology, University Hospital of Basel, Basel, Switzerland

**Keywords:** Intracranial aneurysm, Ruptured intracranial aneurysm, Woven EndoBridge device, Endovascular treatment

## Abstract

**Purpose:**

Although the Woven EndoBridge (WEB) device is increasingly used for the treatment of wide-neck intracranial aneurysms, including in the acute rupture setting, comparative evidence assessing the impact of rupture status remains limited. This study compared angiographic, safety, and clinical outcomes between ruptured and unruptured intracranial aneurysms treated with WEB.

**Methods:**

We conducted a retrospective analysis of prospectively collected data from the multicenter cohort registry WorldWideWEB, including consecutive adult patients with intracranial aneurysms treated with the WEB. Patients were stratified into groups of ruptured and unruptured aneurysms. Propensity score matching was used to balance baseline characteristics between both groups. Retreatment rate was the primary outcome. Secondary outcomes included mRS, safety events (thromboembolic complications) and angiographic outcomes (periprocedurally and last follow-up).

**Results:**

Among 1,220 patients, 342 (28.0%) presented with ruptured aneurysms. Propensity-score-matched analyses revealed no significant difference in thromboembolic complications (11.8% vs. 5.9%, *p* = 0.056), similar periprocedural adequate occlusion (53.3% vs. 53.8%, *p* > 0.9), and similar retreatment rates (11.8% (95% CI 7.8–17.6%) vs. 7.1% (95% CI 4.1–12.0%), *p* = 0.14); however, adequate occlusion at follow-up was lower (82.2% vs. 93.3%, *p* = 0.002) and functional outcomes were worse (mRS ≥ 2 in 34.1% vs. 21.9%, *p* = 0.012) among patients with ruptured aneurysms.

**Conclusion:**

Ruptured aneurysms demonstrated expected inferior follow-up functional and angiographic outcomes when compared with unruptured aneurysms, but no difference in retreatment rate and procedural safety. These findings support WEB as a safe and effective treatment option for appropriately selected ruptured intracranial aneurysms in routine clinical practice.

**Supplementary Information:**

The online version contains supplementary material available at https://doi.org/10.1007/s00234-026-04032-6.

## Introduction

The Woven EndoBridge (WEB) device has become an established endovascular option for the treatment of wide-neck intracranial aneurysms, including selected cases in the acute rupture setting. Compared with techniques such as stent-assisted coiling and flow diversion, its principal advantage is the ability to achieve intrasaccular flow disruption without stent placement in the parent artery, thereby avoiding the need for dual antiplatelet therapy [1].

Since its introduction, the WEB device has been evaluated in multiple prospective trials, and large multicenter registries, demonstrating favorable angiographic outcomes and acceptable safety profiles, especially in wide-neck bifurcation aneurysms [2–5]. Although early investigations focused predominantly on unruptured aneurysms, an increasing number of studies have reported encouraging results in ruptured intracranial aneurysms, including high rates of adequate occlusion, low rebleeding rates, and acceptable complication profiles [6–9]. Meta-analyses and long-term follow-up studies further support the durability of WEB treatment, suggesting that it represents a viable alternative to conventional approaches in appropriately selected ruptured aneurysms [1, 10–12].

Despite these encouraging data, direct comparisons between ruptured and unruptured aneurysms treated with the WEB device remain limited. Many published series of ruptured aneurysms are relatively small or reflect early experience, and differences in baseline clinical status, aneurysm morphology, and treatment strategies may substantially influence angiographic, safety, and functional outcomes [8, 13].

To address this knowledge gap, we conducted a retrospective analysis of prospectively collected data from a large multicenter registry to compare the safety and efficacy profiles of ruptured and unruptured aneurysms. We hypothesized that ruptured aneurysms would demonstrate inferior angiographic and functional outcomes compared with unruptured aneurysms, while retreatment rates and periprocedural safety profiles would remain comparable between groups after adjusting for baseline differences using propensity score matching.

## Methods

### Study design and participants

This study was a retrospective analysis of prospectively collected data from the WorldWideWEB consortium, a multicenter study including 30 academic medical centers across Europe, North America, South America, and Australia [14]. All consecutive adult patients with both ruptured and unruptured intracranial aneurysms, who underwent endovascular treatment using the WEB device, were included. Patients were excluded if there were missing data on rupture status, occlusion rates, long-term follow-up or retreatment records. A total of 1220 of 1612 patients met the inclusion criteria and were included in the final analysis. Aneurysms were then categorized based on their rupture status into two groups of ruptured and unruptured aneurysms. The decision to treat aneurysms with the WEB device was made independently at each participating center by the treating neurointerventionalist, based on aneurysm-specific characteristics including rupture status, anatomical location, size, and neck morphology. Institutional review board approval was obtained at the lead institution, with additional approvals secured at each participating center as required. Given the retrospective design and the relatively low risk and retrospective nature of this study, informed consent was waived. Reporting is in accordance with the STROBE (Strengthening the Reporting of Observational Studies in Epidemiology guidelines for observational studies) statement.

### Data collection

The following information was collected from institutional medical records and entered into the WorldWideWEB Consortium registry: patient demographics (age, sex, smoking status, prior adjunctive treatment), baseline functional impairment (defined as a modified Rankin Scale (mRS) score ≥ 2 prior to treatment), aneurysm-related characteristics (rupture status, anatomical location, size parameters), and treatment characteristics (immediate angiographic occlusion, and immediate Raymond-Roy score). Follow-up data encompassed time since last follow-up, angiographic occlusion outcomes, Raymond-Roy score, and the retreatment rate [15].

### Outcomes

Angiographic outcomes included aneurysm occlusion immediately following the treatment and at follow-up, classified using the Raymond–Roy occlusion scale (adequate occlusion: Raymond-Roy occlusion grade I and II; aneurysm remnant: Raymond-Roy occlusion grade III), as well as categorical assessment of occlusion degree at both time points. Angiographic follow-up was performed using digital subtraction angiography, magnetic resonance angiography, or computed tomography angiography, in accordance with local institutional practice.

Safety outcome comprised the occurrence of thromboembolic complications, including both symptomatic and asymptomatic events, which were identified on digital subtraction angiography by angiographic evidence of intraluminal thrombus formation, impaired contrast filling in previously patent vessels, or new vessel occlusion. Efficacy outcomes included functional status at last follow-up, assessed using mRS, and the rate of aneurysm retreatment.

### Statistical analysis

Continuous variables were compared using t-test or Mann–Whitney *U* test, as appropriate. Categorical variables were compared using the chi-square test or Fisher’s exact test, when applicable. Wilson’s score method was used for the calculation of the 95% confidence intervals (95% CI), unless otherwise specified [16]. Ordinal variables were analyzed using mixed-effects ordinal logistic regression models. Statistical significance was defined as a two-sided *p* value < 0.05. To account for potential confounding between ruptured and unruptured aneurysm groups, propensity score matching was performed adjusted for the variables age, sex, current smoking status, maximum aneurysm diameter, aneurysm neck width, aneurysm width, adjunctive treatment use, and pre-treatment mRS. Patients were matched 1:1 using nearest-neighbor matching without replacement, with an absolute difference between propensity scores of 0.2. A sensitivity analysis repeating the matching with the addition of aneurysm location (bifurcation vs. non-bifurcation) as a covariate was additionally performed. A subgroup analysis was additionally performed restricting the cohort to bifurcation aneurysms only, in whom propensity score matching was repeated using identical parameters to the primary analysis. All statistical analyses were performed using R statistical software, version 4.2.2 (R Foundation for Statistical Computing) [17]. Propensity score matching was conducted using the MatchIt package, and descriptive tables were generated using the tableone and gtsummary packages.

## Results

**Fig. 1:**
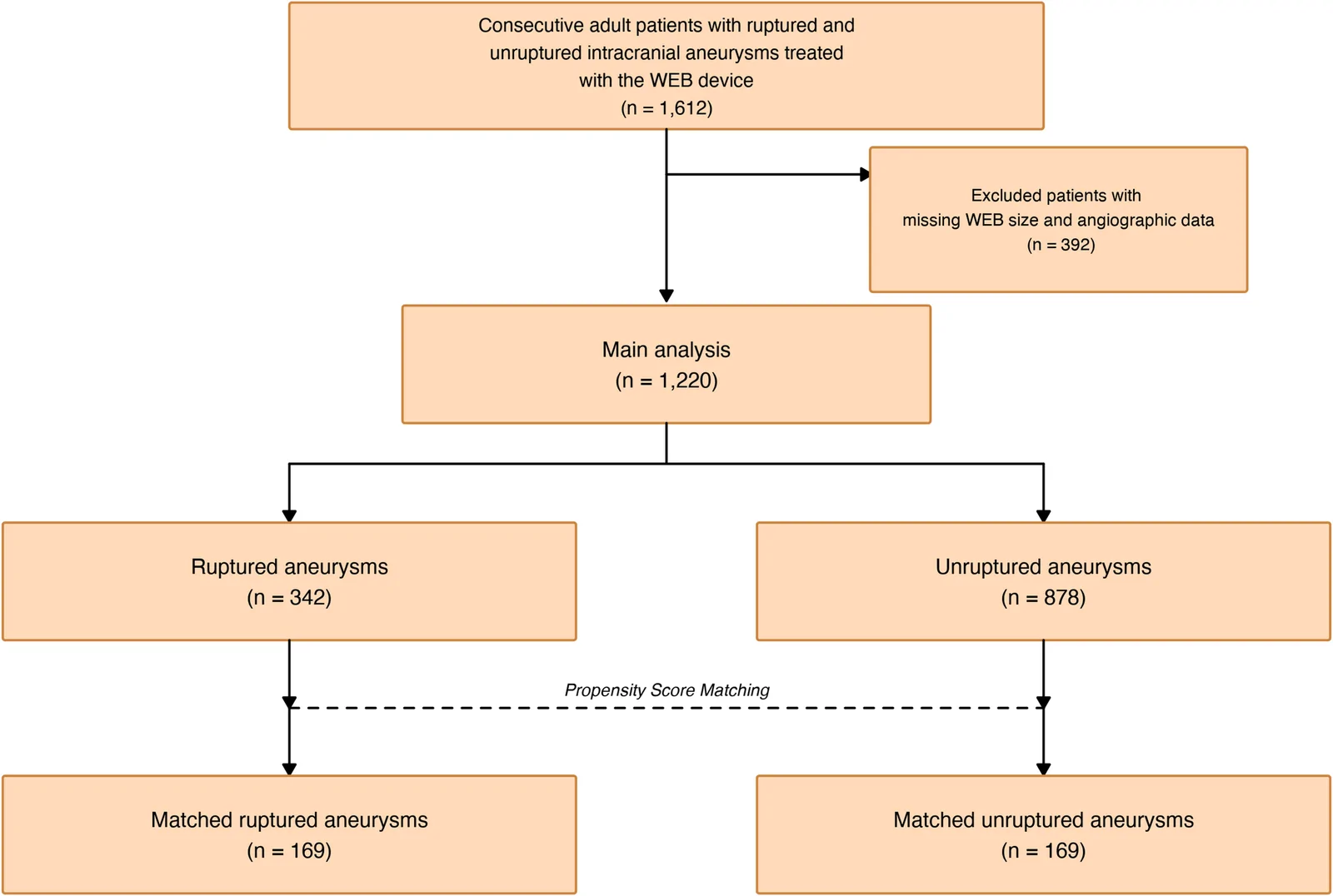
Patient flowchart

A total of 1,220 patients were included, of whom 342/1220 (28.0%) presented with ruptured intracranial aneurysms (Fig. 1). Patients with ruptured aneurysms were younger (median age 56 vs. 61 years), more frequently male, and more often active smokers. The median Hunt–Hess grade among patients with ruptured aneurysms was 2 [IQR 1–3]. Baseline functional impairment (mRS ≥ 2) was significantly more common in the ruptured cohort (98/342 (30.6%) vs. 62/878 (7.5%), *p* < 0.001) (Table 1).

**Table 1:** Baseline patient characteristics

Variable	Overall (*n* = 1220)	Unruptured (*n* = 878)	Ruptured (*n* = 342)	*p*
Male, n (%)	348 (28.5)	223 (25.4)	125 (36.5)	**< 0.001**
Age	59.0 [52.0, 67.5]	61.0 [53.0, 69.0]	56.0 [49.0, 63.0]	**< 0.001**
Current smoking	406 (37.9)	269 (34.8)	137 (45.8)	**0.001**
Hunt-Hess score at presentation				**< 0.001**
0	327 (43.4)	318 (76.3)	9 (2.7)	
1	230 (30.5)	95 (22.8)	135 (40.1)	
2	62 (8.2)	1 (0.2)	61 (18.1)	
3	77 (10.2)	3 (0.7)	74 (22.0)	
4	35 (4.6)	0 (0.0)	35 (10.4)	
5	23 (3.1)	0 (0.0)	23 (6.8)	
Baseline mRS score ≥ 2	160 (14.0)	62 (7.5)	98 (30.6)	**< 0.001**
Prior treatment	89 (7.9)	61 (7.7)	28 (8.5)	0.727
Maximum diameter (mm)	6.5 [5.2, 8.0]	6.5 [5.3, 8.0]	6.3 [5.0, 8.2]	0.424
Characteristics of aneurysms				
Neck (mm)	4.0 [3.0, 5.0]	4.0 [3.1, 5.0]	4.0 [3.0, 4.7]	**0.016**
Width (mm)	5.4 [4.2, 7.0]	5.5 [4.5, 7.0]	5.1 [4.0, 7.0]	**0.017**
Height (mm)	5.7 [4.5, 7.3]	5.7 [4.5, 7.3]	5.8 [4.4, 7.3]	0.875
Location				**< 0.001**
Anterior cerebral artery	412 (33.8)	266 (30.4)	146 (42.7)	
Internal carotid artery	132 (10.8)	119 (13.6)	13 (3.8)	
Vertebrobasilar artery	225 (18.5)	173 (19.7)	52 (15.2)	
Middle cerebral artery	362 (29.7)	274 (31.3)	88 (25.7)	
Posterior cerebral artery	87 (7.1)	44 (5.0)	43 (12.6)	

The majority of procedures were performed without adjunctive devices in both groups (247/266 (92.9%) in ruptured vs. 596/665 (89.6%) in unruptured aneurysms; adjunctive treatment data available in 931/1220 patients). Use of adjunctive treatment did not differ significantly between groups. Periprocedural thromboembolic complications occurred more frequently in ruptured aneurysms (40/342 (11.7%) vs. 48/878 (5.5%), *p* < 0.001). Patients with ruptured aneurysms showed higher rates of immediate adequate occlusion (198/342 (57.9%) vs. 440/878 (50.1%), *p* = 0.017) as well as immediate occlusion status of more than 90% (186/342 (54.4%) vs. 391/878 (44.6%), *p* = 0.003) (Table 2).

**Table 2:** Procedural characteristics, including safety and angiographic outcomes * Adjunctive treatment data available in 931/1220 patients (ruptured: *n* = 266/342, unruptured: *n* = 665/878)

Variable	Overall (*n* = 1220)	Unruptured (*n* = 878)	Ruptured (*n* = 342)	*p*
Periprocedural thromboembolic complications	88 (7.2)	48 (5.5)	40 (11.7)	**< 0.001**
Adjunctive treatment*				0.124
None	843 (90.5)	596 (89.6)	247 (92.9)	
Balloon	15 (1.6)	9 (1.4)	6 (2.3)	
Coiling	31 (3.3)	23 (3.5)	8 (3.0)	
Flow diverter	3 (0.3)	3 (0.5)	0 (0.0)	
Stenting	39 (4.2)	34 (5.1)	5 (1.9)	
Immediate adequate occlusion	638 (52.3)	440 (50.1)	198 (57.9)	**0.017**
Immediate occlusion status more than 90%	577 (47.3)	391 (44.6)	186 (54.4)	**0.003**

Median angiographic follow-up was slightly shorter for ruptured aneurysms (10.0 vs. 11.1 months, *p* = 0.038). Angiographic follow-up data were available in 1190/1220 patients (97.5%; ruptured: *n* = 334/342, unruptured: *n* = 856/878), mRS data in 1209/1220 patients (99.1%; ruptured: *n* = 339/342, unruptured: *n* = 870/878), and retreatment data in 1169/1220 patients (95.8%; ruptured: *n* = 333/342, unruptured: *n* = 836/878). Patients with missing data for specific outcomes were retained in all other analyses. At follow-up, occlusion status of more than 90% was achieved in 289/334 (86.5%) of ruptured and 749/856 (87.5%) of unruptured aneurysms (*p* = 0.722), with no significant difference in adequate occlusion rates (284/334 (85.0%) vs. 760/856 (88.8%), *p* = 0.094). Functional outcomes at follow-up were less favorable in the ruptured cohort, with mRS ≥ 2 observed in 92/339 (27.1%) of patients compared with 87/870 (10.0%) in the unruptured group (*p* < 0.001). Retreatment was more frequently required following rupture (40/333 (12.0%) vs. 62/836 (7.4%), *p* = 0.016) (Table 3).

**Table 3:** Retreatment rates and angiographic outcomes at last follow-up

Variable	Overall (*n* = 1220)	Unruptured (*n* = 878)	Ruptured (*n* = 342)	*p*
Time since last FU (months)	11.0 [5.9, 22.0]	11.1 [6.0, 22.2]	10.0 [3.3, 20.0]	**0.038**
mRS ≥ 2 at follow-up**	179 (14.8)	87 (10.0)	92 (27.1)	**< 0.001**
Occlusion status more than 90%*	1038 (87.2)	749 (87.5)	289 (86.5)	0.722
Adequate occlusion*	1044 (87.7)	760 (88.8)	284 (85.0)	0.094
Retreatment***	102 (8.7)	62 (7.4)	40 (12.0)	**0.016**

* Angiographic follow-up available in 1190/1220 patients (ruptured: *n* = 334, unruptured: *n* = 856). Percentages for occlusion outcomes calculated per patient against this denominator

** mRS data available in 1209/1220 patients (ruptured: *n* = 339, unruptured: *n* = 870)

*** Retreatment data available in 1169/1220 patients (ruptured: *n* = 333, unruptured: *n* = 836)

In a propensity score matched cohort comprising 169 patients with ruptured aneurysms and 169 with unruptured aneurysms, propensity score matching achieved good baseline balance across all variables, with standardized mean difference (SMD) < 0.1 for all covariates after matching (Supplementary Table 1). Angiographic follow-up data were available in 328/338 matched patients (97.0%; ruptured: *n* = 163/169, unruptured: *n* = 165/169), and mRS data in 336/338 matched patients (ruptured: *n* = 167/169, unruptured: *n* = 169/169). Patients without angiographic or mRS data were retained in all other outcome analyses. Ruptured aneurysms demonstrated significantly lower rates of adequate occlusion (134/163 (82.2%) vs. 154/165 (93.3%), *p* = 0.002) and lower rates of occlusion status more than 90% (135/163 (82.8%) vs. 153/165 (92.7%), *p* = 0.006) at follow-up. In addition, patients with ruptured aneurysms showed poorer functional outcomes at follow-up, with a higher proportion having mRS ≥ 2 than in case of unruptured aneurysms (57/167 (34.1%) vs. 37/169 (21.9%), *p* = 0.012). Immediate angiographic outcomes (adequate occlusion 90/169 (53.3%) vs. 91/169 (53.8%), *p* > 0.9; more than 90% occlusion status 81/169 (47.9%) vs. 68/169 (40.2%), *p* = 0.2), retreatment rates (20/169 (11.8%, 95% CI 7.8–17.6%) for ruptured and 12/169 (7.1%, 95% CI 4.1–12.0%) for unruptured aneurysms, *p* = 0.14) and rate of thromboembolic complications (20/169 (11.8%) vs. 10/169 (5.9%), *p* = 0.056) did not differ significantly between groups after matching (Table 4).

**Table 4:** Propensity score matched analysis

Outcome	Overall (*n* = 338)	Unruptured (*n* = 169)	Ruptured (*n* = 169)	*P*-value
Thromboembolic complications	30 (8.9%)	10 (5.9%)	20 (11.8%)	0.056
Raymond-Roy (immediate)				> 0.9
Remnant aneurysm	157 (46.4%)	78 (46.2%)	79 (46.7%)	
Adequate occlusion	181 (53.6%)	91 (53.8%)	90 (53.3%)	
Raymond-Roy (follow-up)*				**0.002**
Remnant aneurysm	40 (12.2%)	11 (6.7%)	29 (17.8%)	
Adequate occlusion	288 (87.8%)	154 (93.3%)	134 (82.2%)	
Occlusion status (immediate)				0.2
Less than 90%	189 (55.9%)	101 (59.8%)	88 (52.1%)	
More than 90%	149 (44.1%)	68 (40.2%)	81 (47.9%)	
Occlusion status (follow-up)*				**0.006**
Less than 90%	40 (12.2%)	12 (7.3%)	28 (17.2%)	
More than 90%	288 (87.8%)	153 (92.7%)	135 (82.8%)	
mRS ≥ 2 at follow-up**				**0.012**
< 2	242 (72.0%)	132 (78.1%)	110 (65.9%)	
≥ 2	94 (28.0%)	37 (21.9%)	57 (34.1%)	
Retreatment	32 (9.5%)	12 (7.1%, 95% CI 4.1 12.0%)***	20 (11.8% 95% CI 7.8–17.6%)***	0.14

*Angiographic follow-up available in 328/338 matched patients (ruptured: *n* = 163, unruptured: *n* = 165)

**mRS data available in 336/338 matched patients (ruptured: *n* = 167, unruptured: *n* = 169)

*** 95% confidence intervals (Wilson score) are provided for the primary outcome (retreatment rate)

In the sensitivity analysis including aneurysm location (bifurcation vs. non-bifurcation) as an additional matching covariate, 167 matched pairs were identified (*n* = 334 total). The primary findings were consistent with the main analysis: adequate occlusion at follow-up remained significantly lower in ruptured aneurysms (133/162 (82.1%) vs. 146/160 (91.2%), *p* = 0.016), and functional outcomes remained worse (55/165 (33.3%) vs. 36/166 (21.7%), *p* = 0.018). Retreatment rates were significantly higher in ruptured aneurysms in this analysis (21/167 (12.6%) vs. 10/167 (6.0%), *p* = 0.038), while occlusion status of more than 90% at follow-up did not reach statistical significance (134/162 (82.7%) vs. 141/160 (88.1%), *p* = 0.2) (Supplementary Table 2). In the bifurcation aneurysm subgroup (133 matched pairs), findings were consistent with the primary analysis: adequate occlusion at follow-up was significantly lower in ruptured aneurysms (106/130 (81.5%) vs. 117/127 (92.1%), *p* = 0.012), functional outcomes were worse (45/131 (34.4%) vs. 25/132 (18.9%), *p* = 0.005), and retreatment rates were significantly higher (17/133 (12.8%) vs. 7/133 (5.3%), *p* = 0.032) (Supplementary Table 3).

## Discussion

In this retrospective analysis of prospectively collected data from the multicenter cohort study, we evaluated whether rupture status influences functional, angiographic, and safety outcomes among patients with intracranial aneurysms treated with the WEB device. By directly comparing ruptured and unruptured aneurysms in a large dataset and adjusting for baseline differences using propensity score matching, our results provide several clinically relevant insights into the performance of the WEB device in the acute rupture setting.

As expected, functional outcomes at follow-up were less favorable in patients with ruptured aneurysms (34.1% with mRS ≥ 2), a finding that persisted after propensity score matching and is most plausibly attributed to the underlying subarachnoid hemorrhage and its complications rather than device-related factors [18].

Immediate angiographic occlusion rates were higher in ruptured than in unruptured aneurysms. This observation is consistent with prior analyses from our multicenter registry, which suggested that acute inflammation and altered hemodynamics in ruptured aneurysms may enhance the prothrombotic effects of the WEB device, potentially promoting more effective early clot formation [19–21]. The paradox of higher immediate but lower follow-up occlusion rates may reflect the transient nature of this prothrombotic environment: in the acute setting of subarachnoid hemorrhage, reduced cerebral blood flow velocity during vasospasm combined with a systemic hypercoagulable state may facilitate early thrombosis within the device, an effect that is unlikely to be sustained once the acute phase resolves. At follow-up, however, ruptured aneurysms demonstrated lower rates of adequate occlusion and a higher prevalence of neck or aneurysm remnants. In the matched analysis, adequate occlusion was achieved in 82.2% of ruptured aneurysms compared with 93.3% of unruptured aneurysms (*p* = 0.002). These findings are comparable to those reported in the CLEVER study, which demonstrated an adequate occlusion rate of 86.5% in ruptured aneurysms at 12 months [9]. Differences between studies are likely explained by aneurysm characteristics and study design, as CLEVER included smaller aneurysms (mean sac width 4.8 mm), a factor known to be associated with more favorable occlusion outcomes [9]. Other studies have reported similarly high rates of adequate occlusion. Van Rooij et al. observed adequate occlusion in 96% of cases at 3-month angiographic follow-up, a finding likely influenced by smaller aneurysm size and shorter follow-up duration [13]. Meta-analyses by Essibayi et al. and Ferreira et al. reported adequate occlusion rates of 87.3% and 83.8%, respectively, at last or 12-month follow-up, results that are consistent with those observed in our cohort [1, 22]. A sensitivity analysis additionally adjusting for aneurysm location (bifurcation vs. non-bifurcation) yielded consistent results, with adequate occlusion remaining significantly lower in ruptured aneurysms (82.1% vs. 91.2%, *p* = 0.016), supporting the robustness of our primary findings.

Periprocedural thromboembolic complications occurred in 11.8% of ruptured aneurysms in our matched cohort. This rate is comparable to those reported in prior studies, including CLEVER (11.7%) and CLARYS (16.7%), and higher than the pooled thromboembolic complication rate of 4.6% reported in the 2025 meta-analysis by Ferreira et al. [1, 9, 23]. In the unmatched analysis, thromboembolic events were more frequent in ruptured than unruptured aneurysms (11.7% vs. 5.5%), consistent with previous reports from the CLARITY and ARETA studies [24, 25]. This may be related to the pro-inflammatory and pro-thrombotic state during SAH, mirroring the slightly higher risk of coil embolization in the ruptured vs. unruptured setting [26]. However, after propensity score matching, this difference was attenuated and no longer statistically significant. Importantly, the majority of aneurysms in both groups were treated without adjunctive devices, underscoring the feasibility of WEB treatment in ruptured aneurysms while avoiding routine stent use in the acute hemorrhagic setting.

The retreatment rate in ruptured aneurysms was 11.8% in the matched analysis and 9.5% overall. These rates are higher than those reported in prospective studies such as CLEVER (1.8% for ruptured aneurysms) but are comparable to meta-analyses [9]. Caroff et al. reported a global retreatment rate of 7.5%, with significantly higher retreatment rates in ruptured compared to unruptured aneurysms (13% vs. 3.9%) [27]. Other studies have reported retreatment rates ranging from 4.2% to 6.8%, while meta-analyses by Essibayi et al. and Ferreira et al. reported retreatment rates of 5.1% and 7.5%, respectively [1, 6, 13, 22]. Notably, in the sensitivity analysis additionally adjusting for bifurcation morphology, retreatment rates reached statistical significance (12.6% vs. 6.0%, *p* = 0.038), suggesting that after accounting for aneurysm morphology, rupture status may independently influence retreatment risk. This finding was further supported by the bifurcation subgroup analysis, in which retreatment rates were similarly significantly higher in ruptured aneurysms (12.8% vs. 5.3%, *p* = 0.032). Taken together, these findings suggest that the higher retreatment rate observed in our cohort likely reflects differences in patient selection, aneurysm complexity, follow-up duration, and retreatment thresholds inherent to real-world multicenter practice rather than inferior angiographic durability of the WEB device.

Despite being one of the largest studies on ruptured aneurysms, treated with WEB device, several limitations warrant consideration when interpreting the findings. First, retrospective design is subject to selection bias, and treatment strategies were determined at the discretion of individual operators. Second, although propensity score matching was used to reduce confounding, residual unmeasured confounders may persist. Third, follow-up duration was relatively short and slightly different between groups, which may influence the assessment of long-term angiographic outcomes. Fourth, detailed antiplatelet therapy data were not systematically recorded in the WorldWideWEB registry. Given the well-documented heterogeneity of antiplatelet strategies in the acute rupture setting and the variability across participating centers, the potential influence of antiplatelet regimen differences on thromboembolic complication rates cannot be fully excluded and should be considered when interpreting the safety outcomes of this study. Finally, with only 32 retreatment events in the matched cohort, this analysis may be underpowered, and the absence of a statistically significant difference between groups should be interpreted with caution. Future prospective studies with standardized follow-up protocols, larger matched cohorts, and mandatory angiographic follow-up of at least 24 months, are warranted to draw definitive conclusions on retreatment rates and to further define the long-term efficacy and safety of the WEB device in ruptured intracranial aneurysms.

## Conclusion

Our propensity score matched analysis demonstrated that ruptured aneurysms show lower rates of adequate occlusion and higher prevalence of remnants at follow-up despite comparable immediate periprocedural angiographic results. Importantly, no significant differences were observed in periprocedural thromboembolic complications or retreatment rates after adjustment. These findings support the WEB device as a viable treatment option for appropriately selected ruptured intracranial aneurysms in routine clinical practice.

## Supplementary Information

Below is the link to the electronic supplementary material.

Supplementary Material 1 (PDF 218 KB)

## Data Availability

No datasets were generated or analysed during the current study.

## References

[1] Ferreira MY, Cardoso LJC, Scarramal JPL, Ribeiro FV, Mora LD, Souza SB et al (2025) Safety and efficacy of the Woven EndoBridge (WEB) device in ruptured intracranial aneurysms: a systematic review and updated meta-analysis. Neuroradiology 67(7):1859–1872. 10.1007/s00234-025-03677-z 40522486

[2] Pierot L, Costalat V, Moret J, Szikora I, Klisch J, Herbreteau D et al (2016) Safety and efficacy of aneurysm treatment with WEB: results of the WEBCAST study. J Neurosurg 124(5):1250–1256. 10.3171/2015.2.Jns142634 26381253

[3] Pierot L, Gubucz I, Buhk JH, Holtmannspötter M, Herbreteau D, Stockx L et al (2017) Safety and efficacy of aneurysm treatment with the WEB: results of the WEBCAST 2 Study. AJNR Am J Neuroradiol 38(6):1151–1155. 10.3174/ajnr.A5178 PMC7960101 28450432

[4] Arthur AS, Molyneux A, Coon AL, Saatci I, Szikora I, Baltacioglu F et al (2019) The safety and effectiveness of the Woven EndoBridge (WEB) system for the treatment of wide-necked bifurcation aneurysms: final 12-month results of the pivotal WEB Intrasaccular Therapy (WEB-IT) Study. J Neurointerv Surg 11(9):924–930. 10.1136/neurintsurg-2019-014815 PMC6824604 30992395

[5] Spelle L, Herbreteau D, Caroff J, Barreau X, Ferré JC, Fiehler J et al (2023) CLinical Assessment of WEB device in Ruptured aneurYSms (CLARYS): 12-month angiographic results of a multicenter study. J Neurointerv Surg 15(7):650–654. 10.1136/neurintsurg-2022-018749 PMC10314057 35882550

[6] Youssef PP, Dornbos ID, Peterson J, Sweid A, Zakeri A, Nimjee SM et al (2021) Woven EndoBridge (WEB) device in the treatment of ruptured aneurysms. J Neurointerv Surg 13(5):443–446. 10.1136/neurintsurg-2020-016405 32719167

[7] van Rooij SB, van Rooij WJ, Peluso JP, Sluzewski M (2018) The Woven EndoBridge (WEB) as primary treatment for unruptured intracranial aneurysms. Interv Neuroradiol 24(5):475–481. 10.1177/1591019918772174 PMC6116134 29768963

[8] Ozpeynirci Y, Braun M, Pala A, Schick M, Schmitz B (2019) WEB-only treatment of ruptured and unruptured intracranial aneurysms: a retrospective analysis of 47 aneurysms. Acta Neurochir (Wien) 161(8):1507–1513. 10.1007/s00701-019-03988-0 31240584

[9] Spelle L, Costalat V, Caroff J, Wodarg F, Fischer S, Herbreteau D et al (2025) Clinical evaluation of WEB 17 device in intracranial aneurysms (CLEVER): 1-year effectiveness results for ruptured and unruptured aneurysms. J Neurointerv Surg 17(8):878–882. 10.1136/jnis-2024-021918 PMC12322418 38991733

[10] Palmisano V, Ganimede MP, Diana F, Lozupone E, Di Stasi C, Burdi N et al (2025) Long-term outcomes of the Woven EndoBridge device for intracranial aneurysms: systematic review and meta-analysis. J Neurointerv Surg. 10.1136/jnis-2025-023799. Epub ahead of print. PMID: 40764017 40764017

[11] Kortman H, van Rooij SBT, Mutlu U, Boukrab I, van Rooij WJ, van der Pol B et al (2023) WEB treatment of ruptured intracranial aneurysms: long-term follow-up of a single-center cohort of 100 patients. AJNR Am J Neuroradiol 44(1):60–64. 10.3174/ajnr.A7748 PMC9835910 36549852

[12] Monteiro A, Lazar AL, Waqas M, Rai HH, Baig AA, Cortez GM et al (2022) Treatment of ruptured intracranial aneurysms with the Woven EndoBridge device: a systematic review. J Neurointerv Surg 14(4):366–370. 10.1136/neurintsurg-2021-017613 34266907

[13] van Rooij SBT, van Rooij WJ, Peluso JP, Sluzewski M, Bechan RS, Kortman HG et al (2017) WEB treatment of ruptured intracranial aneurysms: a single-center cohort of 100 patients. AJNR Am J Neuroradiol 38(12):2282–2287. 10.3174/ajnr.A5371 PMC7963751 28882858

[14] Musmar B, Salim HA, Adeeb N, Aslan A, Aljeradat B, Diestro JDB et al (2024) Treatment of large intracranial aneurysms using the Woven EndoBridge (WEB): a propensity score-matched analysis. Neurosurg Rev 47(1):374. 10.1007/s10143-024-02527-5 PMC11291542 39083214

[15] Fiorella D, Arthur A, Byrne J, Pierot L, Molyneux A, Duckwiler G et al (2015) Interobserver variability in the assessment of aneurysm occlusion with the WEB aneurysm embolization system. J Neurointerv Surg 7(8):591–595. 10.1136/neurintsurg-2014-011251 24984707

[16] Wilson EB (1927) Probable inference, the law of succession, and statistical inference. J Am Stat Assoc 22(158):209–212. 10.2307/2276774

[17] R Core Team (2022) R: A language and environment for statistical computing. Vienna, Austria: R Foundation for Statistical Computing. https://www.R-project.org/. Accessed 21 April 2026

[18] van Gijn J, Kerr RS, Rinkel GJ (2007) Subarachnoid haemorrhage. Lancet 369(9558):306–318. 10.1016/s0140-6736(07)60153-6 17258671

[19] Savarraj JPJ, Parsha K, Hergenroeder GW, Zhu L, Bajgur SS, Ahn S et al (2017) Systematic model of peripheral inflammation after subarachnoid hemorrhage. Neurology 88(16):1535–1545. 10.1212/wnl.0000000000003842 PMC5395070 28314864

[20] de Oliveira Manoel AL, Macdonald RL (2018) Neuroinflammation as a target for intervention in subarachnoid hemorrhage. Front Neurol 9:292. 10.3389/fneur.2018.00292 PMC5941982 29770118

[21] Essibayi MA, Jabal MS, Jamil H, Salim HA, Musmar B, Adeeb N et al (2025) Factors influencing immediate post-angiographic occlusion outcomes in intracranial aneurysms treated with the Woven EndoBridge Device: a multi-center analysis and predictive model from the WorldWideWEB consortium. Neurosurg Rev 49(1):36. 10.1007/s10143-025-03928-w PMC12669363 41326865

[22] Essibayi MA, Lanzino G, Brinjikji W (2021) Safety and efficacy of the Woven EndoBridge Device for treatment of ruptured intracranial aneurysms: a systematic review and meta-analysis. Am J Neuroradiol 42(9):1627–1632. 10.3174/ajnr.A7174 PMC8423055 34117016

[23] Spelle L, Herbreteau D, Caroff J, Barreau X, Ferré J-C, Fiehler J et al (2022) Clinical assessment of WEB device in ruptured aneurYSms (CLARYS): results of 1-month and 1-year assessment of rebleeding protection and clinical safety in a multicenter study. J Neurointerv Surg 14(8):807–814. 10.1136/neurintsurg-2021-017416 PMC9304095 34493578

[24] Pierot L, Cognard C, Anxionnat R, Ricolfi F (2010) Ruptured intracranial aneurysms: factors affecting the rate and outcome of endovascular treatment complications in a series of 782 patients (CLARITY study). Radiology 256(3):916–923. 10.1148/radiol.10092209 20720074

[25] Pierot L, Barbe C, Nguyen HA, Herbreteau D, Gauvrit J-Y, Januel A-C et al (2020) Intraoperative complications of endovascular treatment of intracranial aneurysms with coiling or balloon-assisted coiling in a prospective multicenter cohort of 1088 participants: analysis of recanalization after endovascular treatment of intracranial aneurysm (ARETA) study. Radiology 295(2):381–389. 10.1148/radiol.2020191842 32096707

[26] Park H-K, Horowitz M, Jungreis C, Genevro J, Koebbe C, Levy E et al (2005) Periprocedural Morbidity and Mortality Associated with Endovascular Treatment of Intracranial Aneurysms. Am J Neuroradiol 26(3):506–514 PMID: 15760857 PMC7976477 15760857

[27] Caroff J, Janot K, Soize S, Marnat G, Cortese J, Mihalea C et al (2023) Management of aneurysmal recurrence after Woven EndoBridge (WEB) treatment. J Neurointerv Surg 15(10):939–942. 10.1136/jnis-2022-019645 36288976

