# Supplementary material for: Woven Endobridge device for ruptured vs. unruptured aneurysms: insights from the WorldWideWEB study
Source: Neuroradiology. 2026 May 18;68(6):1593–605. doi: 10.1007/s00234-026-04032-6 (PMC13653826; doi:10.1007/s00234-026-04032-6)
Supplement: Supplementary file 1 [file 234_2026_4032_MOESM1_ESM.pdf]

SUPPLEMENTAL MATERIAL

Woven EndoBridge Device for Ruptured vs. Unruptured Aneurysms: Insights from the WorldWideWEB Study

Journal name: Neuroradiology

Franja Dugar, MD[1]; Muhammed Amir Essibayi, MD, MS[2]; Hamza Adel Salim, MD[3,4]; Basel Musmar, MD[5]; Nimer Adeeb, MD[5]; Mahmoud Dibas, MD[5]; Yan-Lin Li, MBBS MSc[6]; Oktay Algin, MD[7]; Sherief Ghozy, MD[8]; Sovann V. Lay, MD[9]; Adrien Guenego, MD[10]; Leonardo Renieri, MD[11]; Joseph Carnevale, MD[12]; Guillaume Saliou, MD PhD[13]; Panagiotis Mastorakos, MD PhD[14]; Eimad Shotar, MD[15]; Markus Möhlenbruch, MD[16]; Michael Kral, MD[17]; Charlotte Chung, MD PhD[18]; Mohamed M Salem, MD MPH[19]; Ivan Lylyk, MD[20]; Nicole M. Cancelliere MRT(R) MSc[21]; Jose Danilo Bengzon Diestro MD[21]; Paul M. Foreman, MD[22]; Hamza Shaikh, MD[23]; Vedran Župančić, MD[24]; Muhammad U. Hafeez, MD[25]; Joshua Catapano, MD[26]; Muhammad Waqas, MBBS[26]; Giyas Ayberk, MD[27]; Muhammet Arslan, MD[47]; James D. Rabinov, MD[28]; Onur Ergun, MD[48]; Julian Maingard, MD[29]; Clemens M. Schirmer, MD PhD[30]; Mariangela Piano, MD[31]; Anna L. Kühn, MD PhD[32]; Caterina Michelozzi, MD[33]; Robert M. Starke, MD[34]; Ameer Hassan, DO[35]; Mark Ogilvie, MD[36]; Jesse Jones, MD[36]; Waleed Brinjikji, MD[8]; Fulvio Zaccagna, MD, PhD[37]; James T. Grist PhD[37]; Marios Psychogios, MD[38]; Marie T. Nawka, MD[39]; Jens Fiehler, MD[39]; Thi Dan Linh Nguyen-Kim, MD[1]; Bryan Pukenas, MD[19]; Jan-Karl Burkhardt, MD[19]; Thien Huynh, MD MSc[40]; Juan Carlos Martinez-Gutierrez, MD[41]; Sunil A. Sheth, MD[41]; Diana Slawski, MD[42]; Rabih G. Tawk, MD[40]; Benjamin Pulli, MD[42]; Boris Lubicz, MD PhD[10]; Pietro Panni, MD[33]; Ajit S. Puri, MD[32]; Guglielmo Pero, MD[31]; Eytan Raz, MD[18]; Christoph J. Griessenauer, MD[17]; Hamed Asadi, MD PhD[29]; Adnan Siddiqui, MD[26]; Elad I Levy, MD MBA[26]; Neil Haranhalli, MD[2]; David Altschul, MD[2]; Andrew F. Ducruet, MD[26]; Robert W. Regenhardt, MD PhD[28]; Christopher J. Stapleton, MD[28]; Peter Kan, MD[25]; Vladimir Kalousek, MD[23]; Pedro Lylyk, MD[20]; Srikanth Boddu[12]; Jared Knopman, MD[12]; Stavropoula I. Tjoumakaris, MD[14]; Hugo H. Cuellar-Saenz, MD[5]; Pascal M Jabbour, MD[14]; Frédéric Clarençon, MD PhD[15]; Nicola Limbucci, MD[12]; Tufail Patankar, MBBS, PhD, FRCR[43]; Vitor Mendes Pereira MD MSc[21]; Aman B. Patel, MD[28]; Mohammad Almohammad, MD[44]; Andre Kemmling, MD, MHBA[44]; Luca Scarcia, MD[50]; Adam A. Dmytriw, MD MPH MSc[6, 37,45,46]*; Peter B. Sporns MD, MHBA[1,38,39]*, on behalf of the WorldWideWEB Investigators#

Authors' Affiliations:

[1] Department of Radiology, Neuroradiology and Nuclear Medicine, Stadtspital Zurich, Zurich, Switzerland
[2] Department of Neurosurgery, Montefiore Medical Center, Albert Einstein College of Medicine, Bronx, NY, USA
[3] Department of Neuroradiology, MD Anderson Medical Center, Houston, TX 77030, USA
[4] Department of Radiology, Division of Neuroradiology, Johns Hopkins Medical Center, Baltimore, Maryland, USA
[5] Department of Neurosurgery and Interventional Neuroradiology, Louisiana State University, Shreveport, LA, USA
[6] Nuffield Department of Surgical Sciences, Medical Sciences Division, University of Oxford, Oxford, UK
[7] Department of Radiology, Medical Faculty of Ankara University, Ankara, Turkey
[8] Departments of Radiology and Neurosurgery, Mayo Clinic, Rochester, MN, USA
[9] Department of Neuroradiology, Centre Hospitalier de Toulouse, Toulouse, France
[10] Department of Neuroradiology, Hôpital Universitaire Erasme, Bruxelles, Belgium
[11] Department of Neuroradiology, Ospedale Careggi di Firenze, Florence, Italy
[12] Department of Neurosurgery and Neuroradiology, New York Presbyterian Hospital and Weill Cornell School of Medicine, New York, NY, USA
[13] Department of Neuroradiology, Centre Hospitalier Vaudois de Lausanne, Lausanne, Switzerland
[14] Department of Neurosurgery, Thomas Jefferson University Hospital, Philadelphia, PA, USA

[15] Department of Neuroradiology, Hôpital Pitié-Salpêtrière, Paris, France
[16] Department of Neuroradiology, Universitätsklinikum Heidelberg, Heidelberg, Germany
[17] Department of Neurosurgery, Christian Doppler University Hospital & Institute of Neurointervention, Salzburg, Austria
[18] Departments of Radiology & Neurosurgery, NYU Langone Health Center, New York, NY, USA
[19] Department of Neurosurgery, University of Pennsylvania Medical Center, Philadelphia, PA, USA
[20] Department of Neuroradiology, Clínica La Sagrada Familia, Buenos Aires, Argentina
[21] Neurovascular Centre, Divisions of Therapeutic Neuroradiology and Neurosurgery, St. Michael's Hospital, University of Toronto, Toronto, ON, Canada
[22] Department of Neurosurgery, Orlando Health Neuroscience and Rehabilitation Institute, Orlando, FL, USA
[23] Department of Neuroradiology, Clinical Hospital Center 'Sisters of Mercy', Zagreb, Croatia
[24] Department of Neurosurgery, UTMB and Baylor School of Medicine, Houston, TX, USA
[25] Department of Neurosurgery, Barrow Neurological Institute, Phoenix, AZ, USA
[26] Department of Neurosurgery, State University of New York at Buffalo, Buffalo, NY, USA
[27] Department of Neurosurgery, Yildirim Beyazit University, Ankara, Turkey
[28] Neuroendovascular Program, Massachusetts General Hospital, Harvard University, Boston, MA
[29] Department of Neuroradiology, Austin Health, Victoria, Australia
[30] Department of Neurosurgery and Radiology, Geisinger Hospital, Danville, PA, USA
[31] Department of Neuroradiology, Ospedale Niguarda Cà Granda, Milano, Italy
[32] Department of Neuroradiology, UMass Memorial Hospital, Worcester, MA, USA
[33] Department of Neuroradiology, Ospedale San Raffaele, Milano, Italy
[34] Department of Neurosurgery, University of Miami, Miami, FL, USA
[35] Department of Neuroradiology, Valley Baptist Neuroscience Institute, Harlingen, TX, USA
[36] Departments of Neurosurgery and Radiology, University of Alabama at Birmingham, Birmingham, AL, USA
[37] Oxford Centre for Clinical Magnetic Resonance (OCMR), Division of Cardiovascular Medicine, Radcliffe Department of Medicine, University of Oxford, Oxford, UK
[38] Department of Neuroradiology, University Hospital of Basel, Basel, Switzerland
[39] Department of Diagnostic and Interventional Neuroradiology, University Medical Center Hamburg-Eppendorf, Hamburg, Germany
[40] Departments of Radiology and Neurosurgery, Mayo Clinic, Jacksonville, FL, USA
[41] Department of Neuroradiology, University of Texas Health Science Center at Houston, Houston, USA
[42] Department of Radiology, Division of Neuroimaging and Neurointervention, Stanford University School of Medicine
[43] Department of Neuroradiology, Queens Hospital, Barking, Havering and Redbridge University Hospitals NHS Trust, Romford, UK
[44] Department of Neuroradiology, University Hospital Marburg, Marburg, Germany
[45] Neuroendovascular Program, Massachusetts General Hospital & Brigham and Women's Hospital, Harvard University, Boston, MA
[46] Neurointerventional & Neuroanalytics Collaboration (NAN-C), School of Medicine, Toronto Metropolitan University, Toronto, ON
[47] Pamukkale University, Medical Faculty, Radiology Department, Denizli, Turkiye
[48] Uskudar University, Memorial Hospital, Radiology Department, Ankara, Turkiye
[49] Interventional Neurovascular Unit, Careggi University Hospital, Florence, Italy
[50] Department of Neuroradiology, Henri Mondor Hospital, Creteil, France

#A complete list of the WorldWideWEB Investigators is given in the Supplemental Material.
*Both senior authors contributed equally.

Correspondence to: Prof. Dr. Peter B. Sporns, MHBA, peter.sporns@hotmail.de

#WorldWideWEB Investigators

Ali Al Balushi MD[1], Alex Brehm MD[2], Rachel M. McLellan MS[3], Kevin Premat MD[4], Justin E. Vranic MD[3], Jay A. Vachhani MD[5], Vincent M. Tutino PhD[6], Mohamed K. Ibrahim MD[7], Marwa A. Mohammed MD[7], Stéphanie Elens MD[8], Julian Spears MD MSc[9], Erez Nossek MD[10], Monika Killer-Oberfalzer[11], Mohammad A. Aziz-Sultan MD[12], Omer Doron MD PhD[3], Sri Hari Sundararajan MD[1], Yasuaki Inoue MD[3], Shervin Mirshahi MD[3], Cetin Imamoglu MD[12], Ahmet Bayrak MD[12], Assala Aslan MD[13], Christian Swaid MD[13], Miguel Cuellar MD[13]

1 Department of Neurosurgery and Neuroradiology, New York Presbyterian Hospital and Weill Cornell School of Medicine, New York, NY, USA
2 Department of Neuroradiology, University Hospital of Basel, Basel, Switzerland
3 Neuroendovascular Program, Massachusetts General Hospital & Brigham and Women's Hospital, Harvard University, Boston, MA
4 Department of Neuroradiology, Hôpital Pitié-Salpêtrière, Paris, France
5 Departments of Radiology & Neurosurgery, Cooper University Health Care, Camden, NJ, USA
6 Department of Neurosurgery, State University of New York at Buffalo, Buffalo, NY, USA
7 Departments of Radiology and Neurosurgery, Mayo Clinic, Rochester, MN, USA
8 Department of Neuroradiology , Hôpital Universitaire Erasme, Bruxelles, Belgium
9 Department of Neuroradiology, St. Michael's Hospital, Toronto, ON, Canada
10 Departments of Radiology & Neurosurgery, NYU Langone Health Center, New York, NY, USA
11 Department of Neurosurgery, Christian Doppler University Hospital & Institute of Neurointervention, Salzburg, Austria
12 Department of Radiology, Medical Faculty of Ankara University, Ankara, Turkey
13 Department of Neurosurgery and Interventional Neuroradiology, Louisiana State University, Shreveport, LA, USA

Supplementary Tables

Supplementary Table 1: Standardized mean differences for baseline variables before and after propensity score matching

Supplementary Table 2: Sensitivity analysis of propensity score-matched outcomes including aneurysm location (bifurcation vs. non-bifurcation) as an additional matching variable

Supplementary Table 3: Outcomes in propensity score-matched bifurcation aneurysms

Variable	SMD Before Matching	SMD After Matching
Age	0.216	0.039
Sex	0.247	0.060
Current smoking	0.303	0.059
Maximum diameter (mm)	0.137	0.002
Neck width (mm)	0.103	0.009
Aneurysm width (mm)	0.073	0.005
Adjunctive treatment	0.173	0.033
Pre-treatment mRS ≥2	0.469	0.042

Supplementary Table 1: Standardized mean differences for baseline variables before and after propensity score matching

Legend: mRS – modified Rankin scale; SMD - standardized mean difference. SMD <0.1 indicates adequate balance

Outcome	Overall (n = 334)	Unruptured (n = 167)	Ruptured (n = 167)	P-value
**Thromboembolic complications**	32 (9.6%)	12 (7.2%)	20 (12.0%)	0.14
**Raymond-Roy (immediate)**				0.8
Remnant aneurysm	152 (45.5%)	75 (44.9%)	77 (46.1%)	
Adequate occlusion	182 (54.5%)	92 (55.1%)	90 (53.9%)	
**Raymond-Roy (follow-up)***				**0.016**
Remnant aneurysm	43 (13.4%)	14 (8.8%)	29 (17.9%)	
Adequate occlusion	279 (86.6%)	146 (91.2%)	133 (82.1%)	
**Complete occlusion (immediate)**				0.3
Less than 90%	184 (55.1%)	97 (58.1%)	87 (52.1%)	
More than 90%	150 (44.9%)	70 (41.9%)	80 (47.9%)	
**Complete occlusion (follow-up)***				0.2
Less than 90%	47 (14.6%)	19 (11.9%)	28 (17.3%)	
More than 90%	275 (85.4%)	141 (88.1%)	134 (82.7%)	
**mRS > 2****				**0.018**
<2	240 (72.5%)	130 (78.3%)	110 (66.7%)	
≥ 2	91 (27.5%)	36 (21.7%)	55 (33.3%)	
**Retreatment*****	31 (9.3%)	10 (6.0%)	21 (12.6%)	**0.038**

Supplementary Table 2: Sensitivity analysis of propensity score-matched outcomes including aneurysm location (bifurcation vs. non-bifurcation) as an additional matching variable

* Angiographic follow-up available in 322/334 matched patients (ruptured: n=162, unruptured: n=160)

** mRS data available in 331/334 matched patients (ruptured: n=165, unruptured: n=166)

*** Retreatment data available in all 334 matched patients

Outcome	Overall (n = 266)	Unruptured (n= 133)	Ruptured (n = 133)	P-value
**Thromboembolic complications**	28 (10.5%)	10 (7.5%)	18 (13.5%)	0.11
**Raymond-Roy (immediate)**				0.7
Remnant aneurysm	119 (44.7%)	61 (45.9%)	58 (43.6%)	
Adequate occlusion	147 (55.3%)	72 (54.1%)	75 (56.4%)	
**Raymond-Roy (follow-up)***				**0.012**
Remnant aneurysm	34 (13.2%)	10 (7.9%)	24 (18.5%)	
Adequate occlusion	223 (86.8%)	117 (92.1%)	106 (81.5%)	
**Occlusion status (immediate)**				0.3
Less than 90%	141 (53.0%)	75 (56.4%)	66 (49.6%)	
More than 90%	125 (47.0%)	58 (43.6%)	67 (50.4%)	
**Occlusion status (follow-up)***				0.085
Less than 90%	36 (14.0%)	13 (10.2%)	23 (17.7%)	
More than 90%	221 (86.0%)	114 (89.8%)	107 (82.3%)	
**mRS ≥2 at follow-up****				**0.005**
<2	193 (73.4%)	107 (81.1%)	86 (65.6%)	
≥ 2	70 (26.6%)	25 (18.9%)	45 (34.4%)	
**Retreatment**	24 (9.0%)	7 (5.3%)	17 (12.8%)	**0.032**

Supplementary Table 3: Outcomes in propensity score-matched bifurcation aneurysms
* Angiographic follow-up available in 257/266 matched patients (ruptured: n=130, unruptured: n=127)
** mRS data available in 263/266 matched patients (ruptured: n=131, unruptured: n=132)
